# The Influence of Kinematics on Tennis Serve Speed: An In-Depth Analysis Using Xsens MVN Biomech Link Technology

**DOI:** 10.3390/bioengineering11100971

**Published:** 2024-09-27

**Authors:** André V. Brito, Pedro Fonseca, Mário J. Costa, Ricardo Cardoso, Catarina C. Santos, Jaime Fernandez-Fernandez, Ricardo J. Fernandes

**Affiliations:** 1 Centre of Research, Education, Innovation and Intervention in Sport (CIFI2D) and Porto Biomechanics Laboratory (LABIOMEP), Faculty of Sport, University of Porto, 4200-450 Porto, Portugal; up201902341@fade.up.pt (A.V.B.); pedro.labiomep@fade.up.pt (P.F.); mjcosta@fade.up.pt (M.J.C.); davidrcardoso@gmail.com (R.C.); cmsantos@fade.up.pt (C.C.S.); 2 Department of Sport Sciences, Higher Institute of Educational Sciences of the Douro (ISCE-Douro), 4560-708 Penafiel, Portugal; 3 Faculty of Physical Education and Sports Sciences, Universidad de León, 24007 León, Spain; jaime.fernandez@unileon.es; 4 AMRED, Human Movement and Sports Performance Analysis, Universidad de León, 24007 León, Spain

**Keywords:** 3D motion analysis, serve biomechanics, kinematic analysis, inertial measurement system

## Abstract

An inertial measurement system, using a combination of accelerometers, gyroscopes and magnetometers, is of great interest to capture tennis movements. We have assessed the key biomechanical moments of the serve phases and events, as well as the kinematic metrics during the serve, to analyze their influence on serve speed. Eighteen male competitive tennis players, equipped with the inertial measurement units, performed a prolonged serve game consisting of 12 simulated points. Participants were divided into groups A and B in accordance with their positioning above or below the sample average serve speed. Group A (compared with their counterparts) presented with lower back hip adduction and knee flexion, and a higher leftward thoracic tilt during the impact event (−14.9 ± 6.9 vs. 13.8 ± 6.4, 2.8 ± 5.9 vs. 14.3 ± 13.0 and −28.9 ± 6.3 vs. 28.0 ± 7.3°). In addition, group A exhibited higher maximal angular velocities in the wrist and thorax, as well as a lower maximal angular velocity in the back hip than group B (427.0 ± 99.8 vs. 205.4 ± 9.7, 162.4 ± 81.7 vs. 193.5 ± 43.8, 205.4 ± 9.7 vs. 308.3 ± 111.7, 193.5 ± 43.8 vs. 81.1 ± 49.7°/s). The relevant biomechanical differences during the serve were identified, highlighting the changes in joint angles and angular velocities between the groups, providing meaningful information for coaches and players to improve their serve proficiency.

## 1. Introduction

Competitive tennis players should exhibit technical and tactical proficiency, developed mental skills and high levels of physical conditioning to achieve success [1,2]. The effectiveness of these factors is influenced by the match duration and format (i.e., best of three or five sets), as well as the different types of opponents, courts and balls [3]. During prolonged tennis points, players face high physiological demands, characterized by a heart rate of 190–200 beats/minute, lactate up to 8 mmol/L, “very hard” score (17) on ratings of perceived exertion and an exercise intensity corresponding to 80% of their VO_2max._, leading to changes in serve kinematics [4,5,6]. In fact, to prepare players for these efforts, the most recent literature suggests that they need to carry out both endurance and strength training to sustain the high intensity and repetitive movements during a match [7]. 

In tennis, all points start with the serve, which has become the most important action in successful matches [8,9]. This action requires an optimal coordination between both limb and joint movements to generate and transfer forces from the ground to the racket [10]. According to international tennis rules, players must serve alternately from two different positions (the right and left court sides) to the diagonally opposite court, having the ball land within the regulated area [11]. An effective first serve should be a powerful shot to win points directly (achieving an “ace”) or to get to the attacking zone due to a weak return from the opponent (corresponding to ≈72–81% of all of the match) [12]. Thus, appropriate skills, such as well-developed kinetic chain coordination, are essential for performing a powerful and effective serve [13].

The coordination of muscle groups, joint movements and body kinematics is essential to optimize the flat, kick and slice serve [14,15], described by different ball trajectories, speeds and spins [16]. In fact, since the flat serves (usually the first ones) are characterized by more speed and less spin than the kick or slice serves, they tend to be the preferred style. This type of serve produces the straightest ball trajectory after the impact, moving at a shorter distance over the net to land inside the serve area, thus reducing the opponent’s time to react [16,17]. Moreover, tennis serves are classified as foot-up and foot-back serves (back foot close to the front foot during ball release and back foot behind the front foot until lower limb extension, respectively) [18,19]. 

Three published systematic scoping reviews appraised the available evidence on the biophysical characteristics [14] and biomechanical techniques of tennis serves [20,21]. The available literature on serve kinematics provides different perspectives but suggests no consensus on their characterization. However, the most widely accepted studies divide the serve into three distinct phases: preparation, acceleration and follow-through, according to the previous labeling events (e.g., ball release and loading) [10,22]. During the loading event, the dominant upper limb moves behind the body, the thorax is flexed laterally and hyperextended, and the lower limbs are in flexion [23]. Then, the dominant upper limb and racket accelerate, with a fast thoracic rotation and flexion, as well as a vertical jump, generating a high velocity for the ball impact. During the follow-through phase, the upper limbs decelerate with an eccentric action followed by a single lower limb landing [24,25,26]. 

Since serving improvements require a rigorous and detailed understanding of the involved kinematics, 3D motion capture systems are acknowledged as the gold standard for analyzing the serve movement [9,13,24,27,28]. This equipment provides detailed measurements of kinematic and kinetic variables [29,30,31]. Nevertheless, using them in real contexts, like tennis courts, remains a challenge due to the different environmental conditions and movement complexity [32,33]. The 2D video camera is indeed a more accessible and cost-effective type of equipment, facilitating its use in real contexts. However, its analysis is less robust compared to that provided by 3D motion capture systems [34,35]. The Xsens motion capture system is composed of inertial sensors, allowing the capture of movement with minimal constraints in larger spaces [22]. The high cost, regular maintenance and limited accuracy, when compared to other optical systems, could be considered disadvantages [36]. The Xsens has been used with many sports but its application in tennis remains less explored [22,37].

More research is needed to better describe the serve events and phases, including in more ecologically valid environments to help players and coaches to more effectively train and improve their performance [38,39]. In addition, further research will contribute to technological development in sports, providing valuable insights for equipment design and motion analysis. We had the following aims: (i) identify and characterize the key biomechanical moments in serve phases and events; (ii) explore important kinematic metrics during the serve; and (iii) verify the influence of serve kinematics on serve speed. Pertaining to the third aim, we had hypothesized that, in players with a lower serve speed, a lower vertical and horizontal displacement of the center of mass, joint angles and angular velocities would be found in the different events and phases.

## 2. Materials and Methods

### 2.1. Participants

Eighteen male competitive tennis players volunteered to participate in the current study. Their main anthropometric and training background characteristics were as follows: 17.8 ± 2.6 years of age, 178.3 ± 5.9 cm of body height, 66.6 ± 9.6 kg of body mass and 14.2 ± 5.1 h of training/week. The inclusion criteria were established as follows: participants had to be, at least, level 2 (i.e., advanced players), according to the classification [40] of the International Tennis Federation, aged between 15 and 20 years old, in the top 50 national ranking and actively involved in national and international tournaments. Exclusion criteria were players with chronic and acute health issues or injury conditions, history of severe joint or muscle injuries and those currently taking medication that may influence performance. The study was conducted in accordance with the Declaration of Helsinki and approved by the local ethics committee (CEFADE 05.2022), with individual written informed consent forms being signed by all participants or their parents/legal guardians.

### 2.2. Experimental Procedures

**Figure 1:**
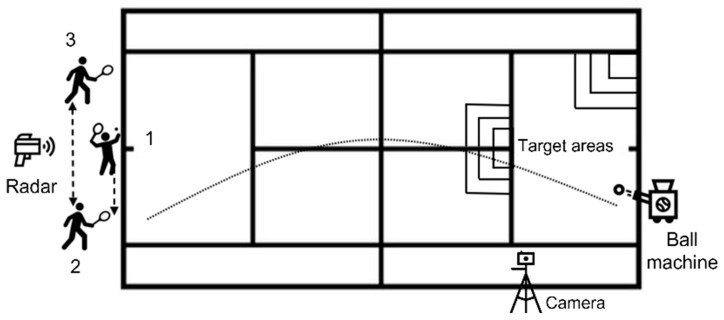
An illustration of the tennis court with the experimental material and the sequence of a simulated point (1, 2 and 3 corresponding to a flat serve, forehand and simulated backhand, respectively).

A prolonged serve game was executed on a grass outdoor court during the morning period in the summer season. After a standardized 15 min warm-up (including mobility exercises, tennis-specific drills and progressive speed serves), players were equipped with an inertial measurement system (Figure 1). Then, they performed a prolonged serve game, of 12 simulated points, each including one flat serve and eight forehands, alternated with eight simulated backhands in square stance position, at maximum intensity (lower limbs parallel to each other and side towards the net; Figure 1). Players used their own rackets, with new tennis balls delivered at a 100 km/h constant speed by a ball machine (Slinger Bag, Slinger LLC, Windsor Mill, MD, USA) placed on the baseline, at 4 s intervals. Serve speed was measured via radar (Stalker Radar Pro II, Richardson, TX, USA) positioned behind the tennis player (Figure 1). This protocol was designed to last ≥40 s (with 30 s intervals between tennis points) to replicate critical moments during a match [39,41]. 

**Figure 2:**
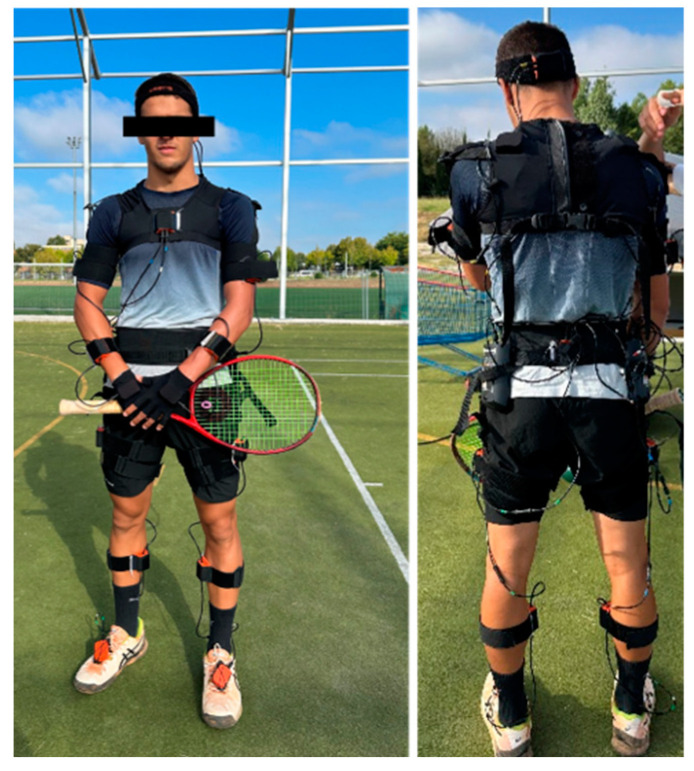
A frontal and back view of the inertial measurement units’ distribution on the participants’ body segments.

Participants were required to wear a previously validated [42] MVN Biomech Link (Xsens Technologies BV, Enschede, The Netherlands) consisting of 17 inertial measurement units, a transmission pack and a battery (Figure 2) [43]. Each unit captures the 6 Degrees of Freedom of the body segment to which it is attached, in real time, at a 100 Hz sampling frequency. The system calibration procedure (i.e., sensor to body alignment and body dimension determination) [44] was performed according to the manufacturer’s instructions with the players assuming an N-pose. During this procedure, the participants kept their upper limbs neutral and parallel to their body, while their lower limbs were kept in full extension and their feet were parallel [45].

### 2.3. Data Analysis

**Figure 3:**
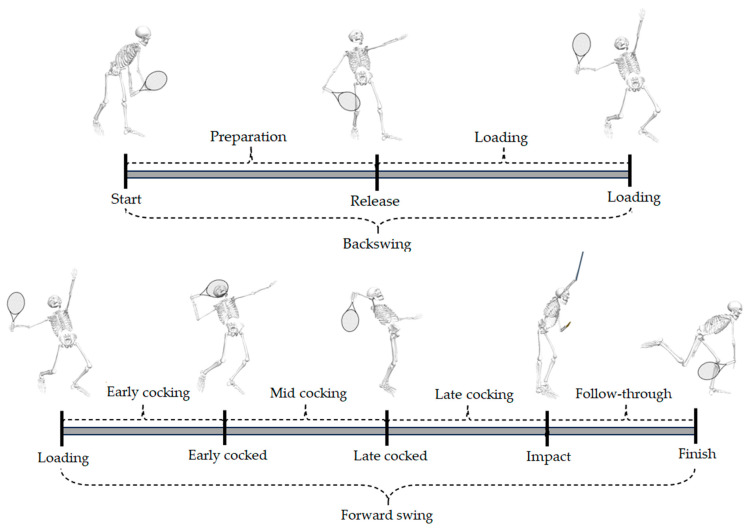
Characterization of tennis serve events and phases.

The first five serves were used for data analysis and serve characterization. The Xsens data was exported in C3D format and imported into Visual 3D software (HAS-Motion, v6, Kingston, ON, Canada), where a 6° of freedom biomechanical model was built. The global and local coordinate systems were created according to Visual3D standards, adapted for the tennis court, with the positive axis in the sagittal right direction (*y*-axis), the frontal plane (*x*-axis) and transverse (*z*-axis) directions. Key serve movement events were identified for each player, according to criteria presented in Table 1. Afterwards, these events were used to determine the serve phases, focusing on the main movements during the serve (e.g., shoulder external and internal rotation; Figure 3) [10,22,46].

Players were grouped based on whether their serve speed was above or below the group mean for serve speed (group A and B, respectively) [15]. For each participant, serve movement was recorded for the entire serve cycle with distinct captures on loading, early and late cocked, and impact events, as well as on the cocking phase (between loading and the impact event) [22]. Event instances and phase duration values during all of the serve movement were calculated [10,13,24,47]. Then, the vertical and horizontal centers of mass displacement were extracted from each event instance, in absolute and relative values (m and % of stature, respectively), regarding the value recorded at the start event. Joint angles, as the angle between the distal and the proximal anatomical segment, were calculated while angular velocity was measured as the change rate of these angles. Finally, the mean and maximum joint angular position and velocity were obtained for each serve phase. 

### 2.4. Statistical Analysis

Data normalcy and equality of variance were assessed using the Shapiro–Wilk test. Normally distributed results were presented as mean and standard deviations [SD], while non-parametric results were shown as median and inter-quartile range (IQR). For variables presenting with a normal distribution, an independent t-test was used to compare the two groups’ mean values of event instances, phase durations, center of mass positions, upper and lower limbs joint angles and angular velocities. The effect size (Cohen’s *d*) was calculated to convey the practical significance of the results, with benchmarks for interpretation as a small (0.2), a moderate (0.5) and a large (0.8) effect size. Otherwise, the Mann–Whitney U test and effect size (r = Z √N, where r, z and N correspond to effect size, value and observation, respectively) was performed to compare all non-parametric variables. Statistical analyses were performed using Statistical Package for the Social Sciences (SPSS 29.0 version, Chicago, IL, USA) for a α = 0.05.

Statistical Parametric Mapping (SPM) analyses were carried out using the open-source spm1d package [48] (version M.0.4.10) on the MATLAB version R2023b (The MathWorks Inc., Portola Valley, CA, USA). An independent t-test was conducted at each time-point along the angular velocity curves to identify statistical differences between the groups (normalized to 101 data points). This analysis involved data normalization, determination of statistical thresholds based on the Random Field Theory and identification of regions where t-values exceeded the critical threshold [49].

## 3. Results

**Figure 4:**
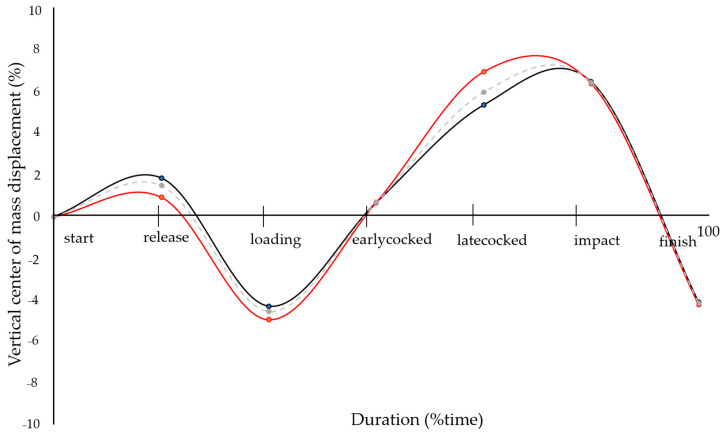
The vertical center of mass displacement from the players’ initial position, during the tennis serve. Red, black and dashed gray lines represent group A, B, and overall, respectively.

The serve event instances, phase durations and center of mass positions during the prolonged serve game are detailed in Table 2, while the center of mass vertical displacement is illustrated in Figure 4. Loading was the most predominant phase during the serve, followed by the preparation, cocking (early, mid and late), and follow-through phases. Late cocking had a greater contribution (*p* = 0.008 and *d* = 1.13) during the serve in group B when compared with group A and the loading event occurred later (*p* = 0.04 and *d* = 0.90) in group B. Regarding their center of mass, group B showed a lower vertical center of mass position (*p* = 0.04 and *d* = −0.89) during the late cocked phase, compared with group A. Additionally, a higher center of mass position was observed during late cocking in group A, while group B reached their highest position at the impact event. No differences were found in the horizontal center of mass or other serve phases and events.

The joint angles for each serve event instance are presented in Table 3 and Table 4 for groups A and B, respectively. The mean serve speed for the entire experimental sample was 139.4 ± 15.7 km/h: 155.0 ± 9.4 km/h for group A and 129.5 ± 10.6 km/h for group B. A comparison of group B with group A revealed the following: (i) lower leftward pelvic tilt (*p* = 0.001 and *d* = 1.31) and lower wrist extension (*p* = 0.03 and *d* = 0.72) at the loading event; (ii) higher shoulder external rotation (*p* = 0.05 and *d* = −0.91), radial wrist deviation (*p* = 0.004 and *d* = −0.89), back hip flexion (*p* = 0.02 and *d* = −1.24) and lower leftward pelvic tilt (*p* = 0.03 and *d* = 1.17) in the early cocking phase; (iii) higher shoulder (*p* = 0.04 and *d* = 0.24) and wrist flexion (*p* = 0.005 and *d* = −1.54), back hip flexion (*p* = 0.05 and *d* = 1.60), knee flexion (*p* = 0.004 and *d* = 0.67), and lower thoracic lateral bending (*p* = 0.004 and *d* = 1.61) during the late cocking phase; and, lastly, (iv) higher back knee flexion (*p* = 0.04 and *d* = 1.04) at the impact event. No differences were observed at the finish event.

**Figure 5:**
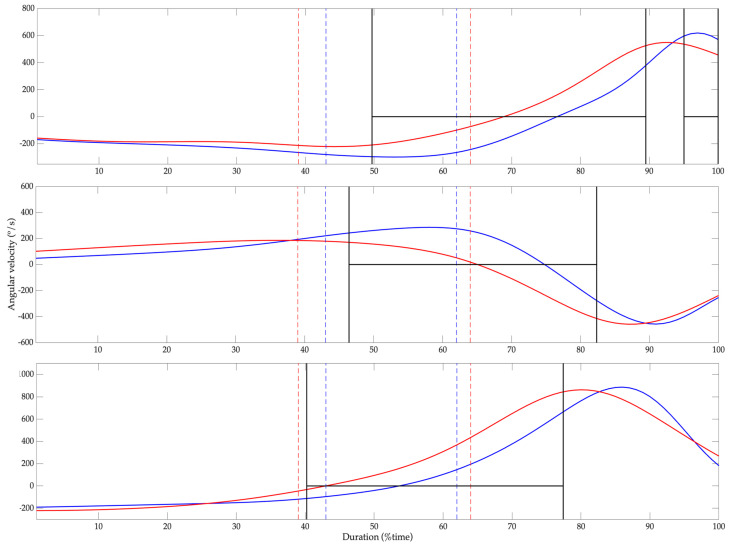
Statistical Parametric Mapping results of Group A and B (solid blue and red lines, respectively) between serve impact and finish event. Shoulder, elbow and wrist angular velocities (first, second and third panel, respectively). The period during which differences between groups with *p* ≤ 0.05 are statistically significant is highlighted by two solid black lines. Early and late cocked events are illustrated by red and blue dashed lines, respectively.

The maximal and minimal joint angular velocities during the cocking phase for the shoulder, elbow, wrist, thorax, pelvis, back and front hip are listed in Table 5. The highest angular velocity was observed at the elbow during flexion and extension. Group B exhibited lower maximal angular velocities during wrist flexion and extension (*p* = 0.03 and *d* = 1.05) and thoracic lateral bending (*p* = 0.01 and *d* = 1.27), as well as a higher maximal angular velocity in the back hip during flexion/extension (*p* = 0.01 and *d* = 0.36), and adduction/abduction (*p* = 0.002 and *d* = 0.56), compared with group A. No differences were registered in the minimal angular velocities. The SPM analysis between the angular velocities of the shoulder, elbow and wrist for groups A and B are presented in Figure 5. Group B showed a lower shoulder, elbow and wrist angular velocity after the early cocked phase (*p* ≤ 0.05), compared with group A, with peak values occurring between 60 and 80% of the serve total duration.

## 4. Discussion

In the current study, we had hypothesized that players with a lower serve speed would have lower vertical and horizontal displacements of their center of mass, joint angles and angular velocities in different events and phases, which were previously identified based on specific biomechanical movement patterns. Our main finding revealed that group A exhibited a greater vertical center of mass position in the late cocking phase compared with group B. Moreover, group B presented with greater back hip adduction and knee flexion, and a lower leftward thoracic tilt during the impact event. Group B also showed lower maximal wrist and thoracic angular velocities, but higher maximal back hip angular velocities compared with group A.

Evaluating the first five serves allowed for a precise and consistent kinematic analysis, without the influence of fatigue, providing useful results for coaches and players [28,38]. In addition, the division of participants into groups A and B was mainly based on their serve speed [50], highlighting its fundamental contribution to the optimization of biomechanical movements (e.g., elbow extension and shoulder external rotation), which provide efficient energy to the ball [13,25]. To characterize the kinematic metrics, it was essential to define some events and phases, assessing absolute values and the curve’s tendencies from the current study and from other published literature, even if the information on this topic remains inconsistent [9,10,51,52]. It was verified that, despite the lower total serve duration observed in this study (1.8 ± 0.2, 2.1 ± 0.2 and 2.1 ± 0.1 s, respectively) [9,34,53], our phase durations presented with similar values when compared with other studies, even though the latter were labeled using different terminology [51,54].

In tennis, a powerful and accomplished serve can significantly influence the point’s success [14]. The players’ center of mass is crucial throughout the serve, especially during the follow-through phase [51]. The lack of stability, a common mistake during the serve, negatively affects the movements after the serve [51,55], particularly during the following shots [25]. Compared with other studies, our players showed similar horizontal displacements of the center of mass in the release, loading, early cocked and finish event phases (−0.4, −0.2, 0.2 and 0.6 m, respectively) [56]. Additionally, this study showed higher and lower vertical displacements of the center of mass at the loading and impact phases (0.88 ± 0.02 and 1.16 ± 0.01 m, respectively), as well as similar values at the finish phase (0.91 ± 0.01 m) [22]. Although the analysis focused on the first five serves to minimize the potential influence of muscle fatigue, this very factor could contribute to the differences in the vertical center of mass values, considering that physical effort continues after the serve [57].

Variations in joint angles can affect the ball speed, spin and precision, impacting the players’ performance and match success [28,53]. As confirmed by other studies [24,35,37,58] lower shoulder external rotation, non-dominant shoulder extension [22,58], elbow, wrist and trunk extension, and knee flexion were identified during loading. Furthermore, our players showed lower shoulder external rotation [24,59,60], higher and lower elbow flexion, and higher [59,60] and similar wrist extension in the cocking phase (early plus late cocked). In addition, the lower values for wrist extension, and higher back knee and thoracic flexion in the impact phase were verified [13,22,61], as well as the lower knee flexion during the finish event [22]. The different levels of player performance and the use of the precise Xsens motion capture system, as opposed to the less advanced devices (e.g., 2D video camera analysis) used in other studies, could explain the discrepancies in the recorded angles.

The shoulder, wrist and thorax provide important kinetic energy, which accumulates and is then transferred through the body into the ball, and directly affect the serve speed [8]. The wrist flexion, shoulder internal rotation, elbow extension, thoracic rotation and flexion, pelvis rotation and back hip extension angular velocities were lower than in other studies (e.g., 2500 ± 511.0, 2400 ± 500.0, 1500.0 ± 200.0, 400.0 ± 30.0, 506.0 ± 69.0, 500.0 ± 40.0 and 230.0 ± 84.3°/s, respectively) [13,27,28,31,47,55,62]. Both shoulder flexion and front hip extension showed higher angular velocities in our study than in others (e.g., 383.0 ± 215.0 and 193.9 ± 76.1°/s, respectively) [31,47,62]. Sample heterogeneity (e.g., age) and anthropometric differences (e.g., height and weight) could affect the range of motion, kinetic energy and muscle strength, since taller players generally have greater reach and leverage, which may allow for faster acceleration of body segments [63,64]. 

Serve speed plays an important role in tennis, influencing the game’s success. Thus, players can win points directly with aces [64,65] or by reducing the opponent’s reaction and their return efficiency [12,37]. Our players presented a lower mean serve speed compared with other studies (189.9 ± 15.1, 184.7 ± 9.5, 149.1 ± 12.1, 164.1 ± 22.5 and 151.4 ± 19.8 km/h) [12,64,66,67,68,69]. Moreover, group B’s mean serve speed presented with a higher value, which contrasted with beginners and intermediate players (95.5 ± 11.8, 101.7 ± 7.4, 101.4 ± 27.6 and 118.0 ± 16.5 km/h, respectively) [35,70,71], whereas group A showed a higher serve speed when compared with competitive players (139.9 ± 20.5 and 135.6 ± 9.2 km/h) and a lower one when examined against professional tennis players (191.0 ± 2.6 km/h) [70,71,72]. The differences in serve speed between the groups can be attributed to the differences in angular velocity during their serve, which suggests that the ability to generate higher angular velocities plays a crucial role in increasing the effectiveness of their serve, compared with players with lower angular velocities [65,73].

The current study revealed significant differences between groups A and B with respect to joint angles and angular velocities during the tennis serve, indicating a direct relationship between these variables and serve speed [20]. Group A exhibited greater external shoulder rotation, and hip and knee flexion during the late cocking phase, suggesting a greater body involvement and kinetic chain coordination, when compared with group B [55]. Group A also showed a higher back hip angular velocity during flexion/extension and adduction/abduction, expressing better kinetic energy efficiency, which is probably associated with their high related joint angles values [17]. In contrast, Group B demonstrated lower wrist flexion/extension and thoracic lateral bending, potentially limiting the energy transfer along the kinetic chain. Consequently, the lower wrist and thoracic angular velocities in group B suggest poor serve technique for generating high ball speed [28]. 

We should point out that this study had some important limitations: (i) the reduced sample size was only 18 players, but a larger number of participants could produce more robust and reliable results; (ii) the age of the sample group resulted in differences in physical and anthropometric development, although the players’ ages were within the inclusion criteria (iii) the players were divided into two groups based on serve speed, despite knowing that other important aspects, for instance precision, could also have been taken into account; and, finally, (iv) wind conditions were not measured and may have affected the serve assessment. For a more comprehensive understanding of serve biomechanics in tennis, future studies with larger samples, different competitive levels and additional performance metrics, such as precision, are recommended.

## 5. Conclusions

This study provides a detailed biomechanical analysis of the tennis serve in competitive players, revealing significant kinematic differences between those with higher and lower serve speeds. Group A, characterized by a higher serve speed, exhibited a higher vertical center of mass during the late cocking phase. Conversely, group B demonstrated greater external shoulder rotation and posterior hip flexion during the cocking phase, along with a reduced leftward pelvic tilt and wrist extension during the loading phase. Additionally, group B displayed lower maximal wrist and thoracic angular velocities compared with group A. These results show distinct biomechanical patterns associated with reduced serve speeds, highlighting the complexity and variability inherent in serve biomechanics. 

This research significantly improves our understanding of serve biomechanics, with important implications for optimizing players’ training and development. Accurate kinematic characterization provides a significant support for coaches to develop targeted and individualized training programs, aimed at improving specific aspects of serve biomechanics. Furthermore, applying inertial measurement systems in ecologically valid environments, such as outdoor tennis courts, increases the training process quality, by enabling continuous monitoring and technique improvement. By incorporating this biomechanical knowledge into training, coaches and players can optimize their serve technique, leading to improved performance in competitive matches.

## Figures and Tables

**Table 1:** Serve criteria definition and study variables, in each anatomical plane, related to tennis serve events and phases.

Stages	Events	Criteria	Segments	Movement Axis and Interpretation
Backswing	Start	The distance between hands increases prior to ball release.	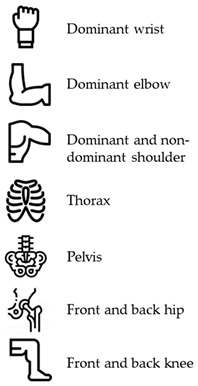	Frontal Plane (*x*-axis): ○ Thorax lateral bending to the left (−and to the right (+); ○ Shoulder, elbow and hip abduction (−) and adduction (+); ○ Wrist radial (−) and ulnar (+) deviation; ○ Pelvis tilt to the left (−) and to the right (+). Sagittal (*y*-axis): ○ Thorax, elbow, wrist, shoulder, hip, knee flexion (+) and extension (−); ○ Pelvis anterior (+) and posterior tilt (−). Transverse (*z*-axis): ○ Thorax rotation to the left (+) and right (−); ○ Shoulder external (−) and internal (+) rotation; ○ Elbow and wrist pronation (+) and supination (−).
	Release	Dominant shoulder with 52° of abduction, 8° of extension or 20° of external rotation.		
	Loading	Maximum knee flexion or minimum center of mass height.		
Forward swing	Early cocked	Maximum shoulder external rotation, without thoracic rotation.		
	Late cocked	Maximum shoulder external rotation caused by inertia of the thoracic rotation.		
	Impact	Maximum elbow extension angle.		
	Finish	Vertical center of mass velocity reaches zero.		

**Table 2:** Group A and B event instances and phase durations of tennis serves.

Phase Duration																
		Preparation (%)			Loading (%)		Cocking (%)						Follow-through (%)		Total serve duration (s)	
							Early		Mid		Late					
Group A		25.3 (6.7)			30.4 (6.0)		10.7 (4.6)		7.8 (3.1)		5.5 (1.2) **		20.0 (3.1)		1.69 (0.14)	
Group B		26.5 (10.2)			32.6 (8.0)		7.9 (4.9)		5.9 (3.8)		7.9 (2.4)		19.0 (3.1)		1.70 (0.18)	
Event		Group A								Group B						
		Instance (%)	Center of mass displacement							Instance (%)		Center of mass displacement				
			Horizontal			Vertical						Horizontal			Vertical	
			%	m		%		m				%		m	%	m
Start		0	0	0		0		1.01 (0.03)		0		0		0	0	0.98 (0.04)
Release		25.1 (6.5)	−1.3 (1.7)	−0.03 (0.2)		0.9 (1.7)		1.03 (0.04)		26.1 (9.0)		−1.8 (3.6)		−0.05 (0.06)	1.8 (0.4)	1.01 (0.04)
Loading		55.5 (4.7)	5.7 (4.9)	0.2 (0.05)		−4.9 (3.5)		0.92 (0.05)		58.9 (2.6) *		10.0 (7.0)		0.1 (0.1)	−4.3 (2.7)	0.90 (0.05)
Cocked	Early	66.0 (5.3)	12.0 (4.3)	0.2 (0.1)		0.6 (2.5)		1.02 (0.05)		67.7 (4.5)		14.0 (7.4)		0.2 (0.09)	0.6 (3.6)	0.99 (0.06)
	Late	70.6 (3.2)	17.2 [4.1]	0.3 (0.1)		6.9 (1.9) *		1.14 (0.02) *		73.2 (2.2)		18.4 (6.8)		0.3 (0.1)	5.3 (1.6)	1.07 (0.05)
Impact		79.8 (3.3)	21.8 (6.4)	0.4 (0.1)		6.4 (2.3)		1.13 (0.03)		81.3 (2.8)		23.6 (7.2)		0.3 (0.1)	6.5 (3.5)	1.09 (0.08)
Finish		100	32.5 (11.3)	0.6 (0.1)		−4.2 (2.5)		0.93 (0.05)		100		31.9 (9.6)		0.5 (0.1)	−4.1 (2.9)	0.91 (0.05)

Symbols * and ** indicate differences with *p* ≤ 0.05 and *p* ≤ 0.01, respectively, between groups A and B.

**Table 3:** Group B joint angles in each tennis serve event.

Events	Motion	Upper Limbs				Lower Limbs				Thorax	Pelvis
		Dominant			Non-Dominant	Front		Back			
		Shoulder	Elbow	Wrist	Shoulder	Knee	Hip	Knee	Hip		
Loading	Frontal	−67.3 (33.6)	NR	4.7 (12.9)	−128.4 (14.1)	NR	1.4 (13.7)	NR	−15.9 (9.9)	13.9 (7.1)	−20.4 (3.7) **
	Sagittal	−10.3 (9.5)	−58.2 (11.4)	0.3 (21.5) *	−32.5 (14.8)	64.3 (10.4)	−11.0 (11.3)	64.2 (10.8)	−11.4 (9.7)	1.9 (11.5)	−0.1 (4.7)
	Transverse	−46.5 (14.9)	NR	NR	NR	NR	−13.1 (4.6)	NR	0.8 (14.9)	−13.4 (8.5)	−109.1 (15.0)
Early cocked	Frontal	−77.4 (17.4)	NR	−4.2 (7.1) **	NR	NR	2.8 (9.2)	NR	−6.7 (7.8)	4.8 (14.0)	−19.0 (1.9) *
	Sagittal	−19.8 (13.7)	−67.2 (11.0)	18.7 (18.8)	NR	39.1 (13.8)	5.7 (7.2)	39.2 (12.3)	9.4 (8.0) *	−9.5 (6.5)	NR
	Transverse	−85.1 (15.3) *	NR	NR	NR	NR	−10.1 (7.5)	NR	1.1 (10.6)	−26.1 (5.7)	−92.1 (16.6)
Late cocked	Frontal	−110.8 (16.5)	NR	−20.1 (17.0)	NR	NR	−15.2 (6.4)	NR	13.1 (3.4)	−26.6 (5.0) **	−13.9 (3.4)
	Sagittal	−29.0 (13.2) *	−48.1 (17.3) **	47.5 (10.6)	NR	18.6 (8.0)	−0.5 (11.0)	4.3 [5.3] **	5.5 (6.8) *	−7.0 (9.5)	NR
	Transverse	−121.8 (19.5)	NR	−26.4 (8.2)	NR	NR	−0.2 (9.5)	NR	−11.6 (7.4)	−13.9 (7.8)	−52.8 (10.0)
Impact	Frontal	−108.6 [9.1]	−8.8 (5.2)	6.1 (6.4)	NR	NR	−23.2 (10.0)	0.03 (1.5)	13.2 (5.8)	−28.9 (6.3)	−16.7 (6.5)
	Sagittal	−28.3 (15.4)	−4.4 (5.2)	18.5 [8.1]	NR	24.8 (11.1)	−28.1 (10.3)	2.8 (5.9) *	−8.5 (9.2)	15.1 (7.4)	17.3 (4.2)
	Transverse	−77.6 (19.1)	59.9 (4.8)	−22.6 (8.5)	NR	NR	−4.3 (12.1)	−6.7 (4.1)	−14.9 (6.9)	3.4 (7.4)	−26.9 (15.3)
Finish	Frontal	18.2 (16.2)	16.3 (7.3)	27.7 [12.6]	NR	NR	−8.4 (12.1)	NR	−14.8 (5.2)	−12.6 (12.1)	−6.3 (9.3)
	Sagittal	−34.9 (10.9)	−27.6 (10.5)	8.6 (8.6)	NR	50.4 (6.5)	−54.6 (13.1)	60.6 (15.7)	−12.0 (12.5)	24.1 (8.4)	25.1 (8.1)
	Transverse	41.1 (21.6)	110.3 (5.0)	25.5 (9.7)	NR	NR	0.2 (10.1)	NR	−11.7 (9.1)	23.7 (10.0)	−1.2 (19.4)

Symbols * and ** p ≤ 0.01 indicate differences with *p* ≤ 0.05 and *p* ≤ 0.01, respectively, between groups A and B. NR (not relevant).

**Table 4:** Group A joint angles in each tennis serve event.

Event	Motion	Upper Limbs				Lower Limbs				Thorax	Pelvis
		Dominant			Non-Dominant	Front		Back			
		Shoulder	Elbow	Wrist	Shoulder	Knee	Hip	Knee	Hip		
Loading	Frontal	−88.8 (29.1)	NR	0.07 (15.7)	−119.5 [15.7]	NR	1.9 (11.9)	NR	−16.7 (14.1)	9.4 (8.8)	−13.0 (6.4)
	Sagittal	−16.7 (15.8)	−58.1 (18.5)	14.5 [10.4]	−30.5 (14.3)	66.9 (9.9)	−18.2 (13.7)	66.7 (7.1)	−16.7 (9.4)	−3.1 (8.6)	−5.4 (5.9)
	Transverse	−74.3 (27.3)	NR	NR	NR	NR	−15.7 (6.5)	NR	2.8 (8.0)	−13.9 (10.5)	−98.3 (13.2)
Early cocked	Frontal	−88.3 (27.3)	NR	−18.0 (18.7)	NR	NR	−1.6 (8.6)	NR	−4.6 (11.6)	1.7 (11.6)	−13.8 (5.7)
	Sagittal	−23.4 (9.8)	−69.7 (8.3)	32.7 (16.2)	NR	44.7 (23.1)	−2.5 (14.9)	42.5 (16.6)	−1.9 (9.8)	−11.0 (12.2)	NR
	Transverse	−104.4 (23.8)	NR	NR	NR	NR	−13.1 (10.0)	NR	3.0 (9.1)	−20.7 [8.8]	−83.1 (21.3)
Late cocked	Frontal	−103.2 (12.6)	NR	−31.7 (13.3)	NR	NR	−12.8 (9.4)	NR	7.8 (9.4)	−16.6 [8.7]	−16.1 (6.8)
	Sagittal	−31.7 (8.0)	−66.6 (6.4)	47.6 (9.7)	NR	20.0 (11.5)	7.4 (8.1)	20.0 (10.0)	9.1 (4.4)	−14.8 (10.2)	NR
	Transverse	−126.2 (13.7)	NR	−18.3 (18.0)	NR	NR	−8.1 (12.0)	NR	−4.9 (10.2)	−16.4 (10.2)	−59.4 (15.2)
Impact	Frontal	−107.7 (11.8)	−6.8 (3.7)	6.2 (6.4)	NR	−1.8 (3.5)	−26.4 (11.4)	1.0 (3.7)	13.8 (6.4)	−28.0 (7.3)	−21.1 (6.8)
	Sagittal	−35.5 (13.5)	−0.1 (8.8)	16.6 (12.2)	NR	27.3 (12.6)	−23.5 (10.3)	14.3 (13.0)	−3.8 (5.8)	10.5 (10.2)	11.7 (7.4)
	Transverse	−72.6 (13.1)	62.8 (14.6)	−22.2 (17.6)	NR	0.4 (4.6)	−10.0 (12.4)	−8.7 (5.0)	−11.2 (7.8)	2.7 (8.8)	−13.8 (21.5)
Finish	Frontal	6.0 (13.5)	23.1 (17.0)	19.4 (6.8)	NR	NR	−7.0 (16.0)	NR	−15.3 (8.9)	−10.0 (5.6)	−14.1 (12.8)
	Sagittal	−40.4 (9.9)	−19.6 (10.8)	11.1 (11.7)	NR	49.4 (10.8)	−54.2 (15.9)	63.0 (10.1)	−16.5 (17.6)	22.5 (7.7)	21.6 (12.1)
	Transverse	31.4 (22.1)	108.3 (19.5)	28.5 (16.7)	NR	NR	−2.3 (13.7)	NR	−11.7 (9.1)	24.5 (9.4)	−15.0 (25.5)

NR (not relevant).

**Table 5:** Group A and B minimal and maximal angular velocities during cocking phase of tennis serve.

Cocking phase	Segments	Motion	Maximal Angular Velocity (°/s)		Minimal Angular Velocity (°/s)	
			Group A	Group B	Group A	Group B
	Shoulder	Sagittal	653.9 (127.4)	629.7 (127.4)	−394.5 (114.7)	−321.3 (103.5)
		Transverse	194.0 (47.3)	188.6 [122.5]	−274.9 (92.1)	−258.8 (151.6)
	Elbow	Sagittal	920.6 (254.9)	1015.2 (122.5)	−245.8 (115.3)	−276.8 (153.1)
	Wrist	Frontal	522.0 (126.2)	554.7 (133.4)	−257.5 (112.1)	−266.8 (89.3)
		Sagittal	427.0 (99.8) *	308.3 (111.7)	−555.6 (169.8)	−592.8 (131.7)
	Thorax	Frontal	162.4 (81.7) *	81.1 (49.7)	−381.7 (68.7)	−342.4 (80.4)
		Sagittal	394.2 (73.2)	344.6 (104.9)	−159.3 (80.8)	−144.7 (80.4)
		Transverse	134.4 (64.6)	113.8 (53.7)	−115.0 (45.4)	−349.7 [103.5]
	Pelvis	Transverse	360.2 (61.2)	407.8 (78.8)	0.1 (23.8)	0.2 (51.8)
	Back hip	Frontal	205.4 (9.7) **	227.8 (77.0)	−84.2 (35.7)	−112.7 (39.8)
		Sagittal	193.5 (43.8) **	240.8 (99.8)	−247.8 (107.1)	−170.8 (77.2)
		Transverse	43.6 (37.4)	66.0 (39.6)	−212.7 (77.2)	−236.8 (55.0)
	Front hip	Frontal	64.2 (49.4)	87.7 (51.6	−205.8 (64.2)	−257.8 (89.1)
		Sagittal	200.6 (67.2)	256.1 (65.1)	−403.1 (49.2)	−342.3 (65.0)
		Transverse	197.2 (43.1)	215.5 (73.6)	−72.6 (44.7)	−87.5 (53.6)

Symbols * and ** indicate differences with *p* ≤ 0.05 and *p* ≤ 0.01 (respectively) between group A and B.

## Data Availability

The dataset generated and analyzed during the current study is available from the corresponding author upon reasonable request.

## References

[1] Lambrich J., Muehlbauer T. (2022). Physical fitness and stroke performance in healthy tennis players with different competition levels: A systematic review and meta-analysis. PLoS ONE.

[2] Kovacs M.S. (2007). Tennis physiology: Training the competitive athlete. Sports Med..

[3] Haake S., Chadwick S., Dignall R., Goodwill S., Rose P. (2001). Engineering tennis-Slowing the game down. Sports Eng..

[4] Morgans L.F., Jordan D.L., Baeyens D.A., Franciosa J.A. (1987). Heart rate responses during singles and doubles tennis competition. Physician Sportsmed..

[5] Fernandez-Fernandez J., Sanz-Rivas D., Fernandez-Garcia B., Mendez-Villanueva A. (2008). Match activity and physiological load during a clay-court tennis tournament in elite female players. J. Sports Sci..

[6] Mendez-Villanueva A., Fernandez-Fernandez J., Bishop D., Fernandez-Garcia B., Terrados N. (2007). Activity patterns, blood lactate concentrations and ratings of perceived exertion during a professional singles tennis tournament. Br. J. Sports Med..

[7] Deng N., Soh K.G., Abdullah B., Huang D., Sun H., Xiao W. (2023). Effects of physical training programs on female tennis players’ performance: A systematic review and meta-analysis. Front. Physiol..

[8] Girard O., Micallef J.P., Millet G.P. (2005). Lower-limb activity during the power serve in tennis: Effects of performance level. Med. Sci. Sports Exerc..

[9] Reid M., Whiteside D., Elliott B. (2010). Effect of skill decomposition on racket and ball kinematics of the elite junior tennis serve. Sports Biomech..

[10] Kovacs M., Ellenbecker T. (2011). An 8-Stage Model for Evaluating the Tennis Serve:Implications for Performance Enhancement and Injury Prevention. Sports Health.

[11] (2023). I.T.Federation. ITF Rules of Tennis. https://www.itftennis.com/media/7221/2023-rules-of-tennis-english.pdf.

[12] Keller M., Kuhn Y.-A., Lüthy F., Taube W. (2021). How to Serve Faster in Tennis: The Influence of an Altered Focus of Attention and Augmented Feedback on Service Speed in Elite Players. J. Strength Cond. Res..

[13] Fett J., Oberschelp N., Vuong J.L., Wiewelhove T., Ferrauti A. (2021). Kinematic characteristics of the tennis serve from the ad and deuce court service positions in elite junior players. PLoS ONE.

[14] Brito A.V., Afonso J., Silva G., Fernandez-Fernandez J., Fernandes R.J. (2024). Biophysical characterization of the tennis serve: A systematic scoping review with evidence gap map. J. Sci. Med. Sport.

[15] Elliott B. (2006). Biomechanics and tennis. Br. J. Sports Med..

[16] Elliott B. (1983). Spin and the power serve in tennis. J. Hum. Mov. Stud..

[17] Chow J.W., Carlton L.G., Lim Y.T., Chae W.S., Shim J.H., Kuenster A.F., Kokubun K. (2003). Comparing the pre- and post-impact ball and racquet kinematics of elite tennis players’ first and second serves: A preliminary study. J. Sports Sci..

[18] Liang Z., Wu J., Yu J., Ying S., Liu Z., Zhang Y., Gu Y., Li J. (2023). Comparison and analysis of the biomechanics of the lower limbs of female tennis players of different levels in foot-up serve. Front. Physiol..

[19] Martin C. (2015). Should players serve using the foot-up or foot-back technique?. ITF Coach. Sport Sci. Rev..

[20] Lambrich J., Muehlbauer T. (2023). Biomechanical analyses of different serve and groundstroke techniques in tennis: A systematic scoping review. PLoS ONE.

[21] Vacek J., Vagner M., Cleather D.J., Stastny P. (2023). A Systematic Review of Spatial Differences of the Ball Impact within the Serve Type at Professional and Junior Tennis Players. Appl. Sci..

[22] Brocherie F., Dinu D. (2022). Biomechanical estimation of tennis serve using inertial sensors: A case study. Front. Sports Act. Living.

[23] Tubez F., Schwartz C., Croisier J.L., Brüls O., Denoël V., Paulus J., Forthomme B. (2021). Evolution of the trophy position along the tennis serve player’s development. Sports Biomech..

[24] Elliott B., Fleisig G., Nicholls R., Escamilia R. (2003). Technique effects on upper limb loading in the tennis serve. J. Sci. Med. Sport.

[25] Fleisig G., Nicholls R., Elliott B., Escamilla R. (2003). Kinematics used by world class tennis players to produce high-velocity serves. Sports Biomech..

[26] Bahamonde R. Joint Power Production During the Flat and Slice Tennis Serves. Proceedings of the 15 International Symposium on Biomechanics in Sports (1997).

[27] Tubez F., Forthomme B., Croisier J.L., Brüls O., Denoël V., Paulus J., Schwartz C. (2019). Inter-Session Reliability of the Tennis Serve and Influence of the Laboratory Context. J. Hum. Kinet..

[28] Martin C., Bideau B., Delamarche P., Kulpa R. (2016). Influence of a Prolonged Tennis Match Play on Serve Biomechanics. PLoS ONE.

[29] King E., Richter C., Franklyn-Miller A., Daniels K., Wadey R., Jackson M., Moran R., Strike S. (2018). Biomechanical but not timed performance asymmetries persist between limbs 9 months after ACL reconstruction during planned and unplanned change of direction. J. Biomech..

[30] Marques J.B., Paul D.J., Graham-Smith P., Read P.J. (2020). Change of Direction Assessment Following Anterior Cruciate Ligament Reconstruction: A Review of Current Practice and Considerations to Enhance Practical Application. Sports Med..

[31] Reid M., Giblin G., Whiteside D. (2014). A kinematic comparison of the overhand throw and tennis serve in tennis players: How similar are they really?. J. Sports Sci..

[32] Suzuki Y., Ae M., Takenaka S., Fujii N. (2014). Comparison of support leg kinetics between side-step and cross-step cutting techniques. Sports Biomech..

[33] Condello G., Kernozek T.W., Tessitore A., Foster C. (2016). Biomechanical Analysis of a Change-of-Direction Task in Collegiate Soccer Players. Int. J. Sports Physiol. Perform..

[34] Gillet E., Leroy D., Thouvarecq R., Stein J.F. (2009). A notational analysis of elite tennis serve and serve-return strategies on slow surface. J. Strength Cond. Res..

[35] Sgrò F. Analysis of Knee Joint Motion in Tennis Flat Serve Using Low-Cost Technological Approach. Proceedings of the 2013 International Workshop on Computer Science in Sports.

[36] García-de-Villa S., Casillas-Pérez D., Jiménez-Martín A., García-Domínguez J.J. (2023). Inertial sensors for human motion analysis: A comprehensive review. IEEE Trans. Instrum. Meas..

[37] Hornestam J.F., Souza T.R., Magalhães F.A., Begon M., Santos T.R.T., Fonseca S.T. (2021). The Effects of Knee Flexion on Tennis Serve Performance of Intermediate Level Tennis Players. Sensors.

[38] Rota S., Morel B., Saboul D., Rogowski I., Hautier C. (2014). Influence of fatigue on upper limb muscle activity and performance in tennis. J. Electromyogr. Kinesiol..

[39] Brito A., Carvalho D., Fonseca P., Monteiro A.S., Fernandes A., Fernandez-Fernandez J., Fernandes R. (2022). Shoulder Torque Production and Muscular Balance after Long and Short Tennis Points. Int. J. Environ. Res. Public Health.

[40] Im S., Lee C.-H. (2023). World Tennis Number: The new gold standard, or a failure?. ITF Coach. Sport Sci. Rev..

[41] Reid M., Duffield R., Dawson B., Baker J., Crespo M. (2008). Quantification of the physiological and performance characteristics of on-court tennis drills. Br. J. Sports Med..

[42] Nijmeijer E., Heuvelmans P., Bolt R., Gokeler A., Otten E., Benjaminse A. (2023). Concurrent validation of the Xsens IMU system of lower-body kinematics in jump-landing and change-of-direction tasks. J. Biomech..

[43] Karatsidis A., Bellusci G., Schepers H.M., De Zee M., Andersen M.S., Veltink P.H. (2017). Estimation of Ground Reaction Forces and Moments During Gait Using Only Inertial Motion Capture. Sensors.

[44] De Leva P. (1996). Adjustments to Zatsiorsky-Seluyanov’s segment inertia parameters. J. Biomech..

[45] Roetenberg D., Luinge H., Slycke P. (2009). Xsens MVN: Full 6DOF human motion tracking using miniature inertial sensors. Xsens Motion Technol. BV Tech. Rep..

[46] Gillet B., Begon M., Berger-Vachon C., Rogowski I. (2017). Kinematics of Shoulder joints during tennis serve in young female athletes: Influence of history of shoulder pain. ISBS Proc. Arch..

[47] Wagner H., Pfusterschmied J., Tilp M., Landlinger J., von Duvillard S.P., Müller E. (2014). Upper-body kinematics in team-handball throw, tennis serve, and volleyball spike. Scand. J. Med. Sci. Sports.

[48] Pataky T.C. (2012). One-dimensional statistical parametric mapping in Python. Comput. Methods Biomech. Biomed. Engin..

[49] Brett M., Penny W., Kiebel S. (2004). An Introduction to Random Field Theory. Human Brain Function.

[50] Jacquier-Bret J., Gorce P. (2024). Kinematics of the Tennis Serve Using an Optoelectronic Motion Capture System: Are There Correlations between Joint Angles and Racket Velocity?. Sensors.

[51] Talaat S., Attaallah M. (2015). Kinematic Analysis of the whole body Center of Gravity Trajectory and Time Structure of the Tennis Serve Performance. J. Appl. Sports Sci..

[52] Whiteside D., Elliott B., Lay B., Reid M. (2013). A kinematic comparison of successful and unsuccessful tennis serves across the elite development pathway. Hum. Mov. Sci..

[53] Reid M., McMurtrie D., Crespo M. (2010). Title: The relationship between match statistics and top 100 ranking in professional men’s tennis. Int. J. Perform. Anal. Sport..

[54] Kibler W.B., Chandler T.J., Shapiro R., Conuel M. (2007). Muscle activation in coupled scapulohumeral motions in the high performance tennis serve. Br. J. Sports Med..

[55] López de Subijana C., Navarro E. (2010). Kinetic energy transfer during the Tennis serve. Biol. Sport..

[56] Jamkrajang P., Robinson M.A., Limroongreungrat W., Vanrenterghem J. (2017). How do tennis players control their balance during the serve?. ISBS Proc. Arch..

[57] Tornero-Aguilera J., Jimenez Morcillo J., Rubio-Zarapuz A., Clemente-Suárez V. (2022). Central and Peripheral Fatigue in Physical Exercise Explained: A Narrative Review. Int. J. Environ. Res. Public Health.

[58] Bingül B.M., Aydin M., Bulgan Ç., Gelen E., Özbek A. (2016). Upper extremity kinematics of flat serve in tennis. S. Afr. J. Res. Sport Phys. Educ. Recreat..

[59] Fleisig G., Nicholls R., Escamilla R., Elliott B. (2002). Kinematics and Kinetics of the High Velocity Tennis Serve. Med. Sci. Sports Exerc..

[60] Touzard P., Kulpa R., Bideau B., Montalvan B., Martin C. (2019). Biomechanical analysis of the “waiter’s serve” on upper limb loads in young elite tennis players. Eur. J. Sport Sci..

[61] Göktepe A., Ak E., Söğüt M., Karabörk H., Korkusuz F. (2009). Joint angles during successful and unsuccessful tennis serves kinematics of tennis serve. Eklem Hastalik. Cerrahisi.

[62] Connolly M., Middleton K., Spence G., Cant O., Reid M. (2021). Effects of Lumbar Spine Abnormality and Serve Types on Lumbar Kinematics in Elite Adolescent Tennis Players. Sports Med.-Open.

[63] Sánchez-Pay A., Ramón-Llin J., Martínez-Gallego R., Sanz D., Sánchez-Alcaraz Martínez B., Frutos S. (2021). Fitness testing in tennis: Influence of anthropometric characteristics, physical performance, and functional test on serve velocity in professional players. PLoS ONE.

[64] Fett J., Ulbricht A., Ferrauti A. (2020). Impact of Physical Performance and Anthropometric Characteristics on Serve Velocity in Elite Junior Tennis Players. J. Strength Cond. Res..

[65] Colomar J., Corbi F., Brich Q., Baiget E. (2022). Determinant Physical Factors of Tennis Serve Velocity: A Brief Review. Int. J. Sports Physiol. Perform..

[66] Fernandez-Fernandez J., Nakamura F.Y., Moreno-Perez V., Lopez-Valenciano A., Del Coso J., Gallo-Salazar C., Barbado D., Ruiz-Perez I., Sanz-Rivas D. (2019). Age and sex-related upper body performance differences in competitive young tennis players. PLoS ONE.

[67] Hayes M.J., Spits D.R., Watts D.G., Kelly V.G. (2021). Relationship Between Tennis Serve Velocity and Select Performance Measures. J. Strength Cond. Res..

[68] Bilić Z., Martić P., Barbaros P., Sinković F., Novak D. (2024). Neuromuscular Fitness Is Associated with Serve Speed in Young Female Tennis Players. Sports.

[69] Eriksrud O., Ghelem A., Henrikson F., Englund J., Brodin N. (2018). Upper and lower body power tests predict serve performance in national and international level male tennis players. Sport Perform. Sci. Rep..

[70] Mavvidis A., Manousaridou A., Grivas N., Evagelidis T., Laios A. (2014). The effectiveness of serve in tennis depending on the placement of palm across the racket grip inwards or outwards. J. Phys. Educ. Sport.

[71] Söğüt M. (2017). A Comparison of Serve Speed and Motor Coordination between Elite and Club Level Tennis Players. J. Hum. Kinet..

[72] Carboch J., Süss V. (2015). Toss differences between the slice serve and the kick serve in tennis. Acta Gymnica.

[73] Lertwonghattakul T., Sriramatr S. (2023). Analysis of kinetic chain mechanism affecting energy flow in kick topspin tennis serve in elite and amateur tennis players. Ann. Appl. Sport Sci..

